# Modelling of Land Use/Cover and LST Variations by Using GIS and Remote Sensing: A Case Study of the Northern Pakhtunkhwa Mountainous Region, Pakistan

**DOI:** 10.3390/s22134965

**Published:** 2022-06-30

**Authors:** Akhtar Rehman, Jun Qin, Sedra Shafi, Muhammad Sadiq Khan, Siddique Ullah, Khalid Ahmad, Nazir Ur Rehman, Muhammad Faheem

**Affiliations:** 1 School of Environmental Studies, China University of Geosciences, Wuhan 430074, China; akhtarktk17@gmail.com (A.R.); faheem@cug.edu.cn (M.F.); 2 School of Geosciences and Information Physics, Central South University, Changsha 410083, China; sedrashafi@csu.edu.cn; 3 State Key Laboratory of Urban and Regional Ecology, Research Center for Eco-Environmental Sciences, Chinese Academy of Sciences, Beijing 100085, China; khan_st@rcees.ac.cn; 4 University of Chinese Academy of Sciences, Beijing 100049, China; 5 Department of Environmental Science, COMSATS University Islamabad (CUI), Abbottabad Campus, Abbottabad 22060, Pakistan; siddiqullah142@gmail.com (S.U.); khalid.taxonomist@gmail.com (K.A.); 6 Department of Geology, Khushal Khan Khattak University, Karak 27200, Pakistan; nazirktk@yahoo.com

**Keywords:** radiative transfer method, modelling, cellular automata, lower mountainous region

## Abstract

Alteration in Land Use/Cover (LULC) considered a major challenge over the recent decades, as it plays an important role in diminishing biodiversity, altering the macro and microclimate. Therefore, the current study was designed to examine the past 30 years (1987–2017) changes in LULC and Land Surface Temperature (LST) and also simulated for next 30 years (2047). The LULC maps were developed based on maximum probability classification while the LST was retrieved from Landsat thermal bands and Radiative Transfer Equation (RTE) method for the respective years. Different approaches were used, such as Weighted Evidence (WE), Cellular Automata (CA) and regression prediction model for the year 2047. Resultantly, the LULC classification showed increasing trend in built-up and bare soil classes (13 km^2^ and 89 km^2^), and the decreasing trend in vegetation class (−144 km^2^) in the study area. In the next 30 years, the built-up and bare soil classes would further rise with same speed (25 km^2^ and 36.53 km^2^), and the vegetation class would further decline (−147 km^2^) until 2047. Similarly for LST, the temperature range for higher classes (27 -< 30 °C) increased by about 140 km^2^ during 1987–2017, which would further enlarge (409 km^2^) until 2047. The lower LST range (15 °C to <21 °C) showed a decreasing trend (−54.94 km^2^) and would further decline to (−20 km^2^) until 2047 if it remained at the same speed. Prospective findings will be helpful for land use planners, climatologists and other scientists in reducing the increasing LST associated with LULC changes.

## 1. Introduction

One of the foremost environmental problems influencing the natural ecosystem is the increasing urbanization worldwide. Because of various socio-economic factors, urbanization triggers migration, altering the global urban pattern. These trends of increasing urbanization have both positive and negative consequences. The negative impacts include health issues, infrastructure burden and environmental degradation, while the positive impacts include job opportunities and improved quality of life [1,2,3,4]. Rapid urbanization also caused significant changes in biodiversity, habitat, natural landscaping, geography, and biophysical climate. According to a World Bank report, 3.5 billion people will live in urban areas by 2030: that will be 60% of the total global population [5]. The rising pattern in global urbanization has provoked numerous researchers to find out the impacts of human actions on metropolitan thermal surroundings, i.e., LST and UHIs [6]. In the previous centuries, due to human interference the LULC [7] notably affected the terrestrial ecosystem at the local, regional and global level [8,9,10,11], thus affecting the overall environment.

The LST is the surface radiation level affected by topographical situations, landscaping configuration, natural vegetation and urban extension [12,13,14]. Increasing tendency of surface temperature in the region of built-up and bare soil are mainly due to impervious levels to come up with formation of UHIs [15]. The LST depends on LULC in tropical and sub-tropical municipal regions where built-up and bare soil have a higher LST than the arid environment [16,17]. The bare earth gains high solar radiation, thus leading to differences in the LST [18]. The alterations in LULC and its impact on LST has been broadly examined [3,19] and it has also been observed that human are responsible for the rising trend in LST for replacing vegetation areas with built-up regions, as the formations of “UHIs” thus brings changes in LULC [20,21].The LULC is made up of several components, which are involved in energy transferformation between the Earth’s surface and the atmosphere [22]. LULC changes have an impact on this energy transformation [6,23].

Simulation models are important to investigate the changes in LULC [24,25]. LULC-based studies provide a significant map for sound decisions in the area of land use. Land Use Scenarios Dynamic model (LUSD) identified the scenario simulation of land use to join “bottom–up” and “top–down” approaches. Natural and manmade factors at different levels are responsible for LULC change [26]. The LULC studies have also extensively been done with remote sensing approaches [27,28]. Different research has reported that remote sensing satellite data is assistive to determine the relation between LST and LULC patterns [29,30]. Numerous methods and algorithms can be used for LULC modeling but the common LULC prediction models are Markov Chain [31], Artificial Neural Network (ANN) model [32] and CA model [33]. The combination of models such as CA-stochastic and CA-ANN forecasted multi-dimensional alteration and provide appropriate outcome [9,34]. Many researchers focused only on LULC changes in urban and rural mountainous areas of Pakistan [35,36,37,38].There has been very little research conducted on LULC changes and their impact on associated LST patterns in the mountainous area located near the Indus river basin area of Northern, Pakistan. The current study employed CA-ANN model for LULC simulation for the year 2047. The model accuracy was validated by Kappa variation using simulated and observed LULC map [35].

The mountainous areas of northern Pakhtunkhwa valley Pakistan are very crucial for their beauty and natural assets. this area is home to the largest fill-kind dam in the world, constructed on the Indus River close to Terbella area; this is also the world’s second largest dam regarding reservoir potential—14.3 billion cubic meters with an installed capacity of 4888 MW; 6298 MW (max). The dam regulates the flows of Indus basin for irrigation, hydroelectricity production and flood management by sustaining snowmelt and River Indus monsoon flow. Moreover, the construction of China and Pakistan Economic Corridor (CPEC) project is very susceptible to link the road between China and Pakistan, which will prove a very useful route for easy travelling and business. Landscaping alteration, natural habitat, even a massive flow of people from other regions in the previous few decades might have induced LULC and LST changes. Although, changes in topography and their consequences on LST and LULC are not identified and such research work has not been designed yet on the subsequent area, which will be a novel contribution for research bank and environment worldwide. This shortage of knowledge created a problem for urban planners, climatologists, researchers and policy makers to manage a sound plan for indigenous communities. For that reason, the present novel project in the lower mountainous area of northern Pakhtunkhwa valley, Pakistan was designed to plan the present LULC and LST changes and its upcoming prediction based on the following objectives:

(a) Investigated LULC changes and LST pattern from 1987–2017 using moderate resolution Landsat data (Landsat 5 TM, Landsat 7 ETM+ and Landsat 8 OLI).
(b) To simulate changes in LULC and LST using regression analysis and CA-ANN model until 2047.

## 2. Materials and Methods

### 2.1. Study Area

**Figure 1:**
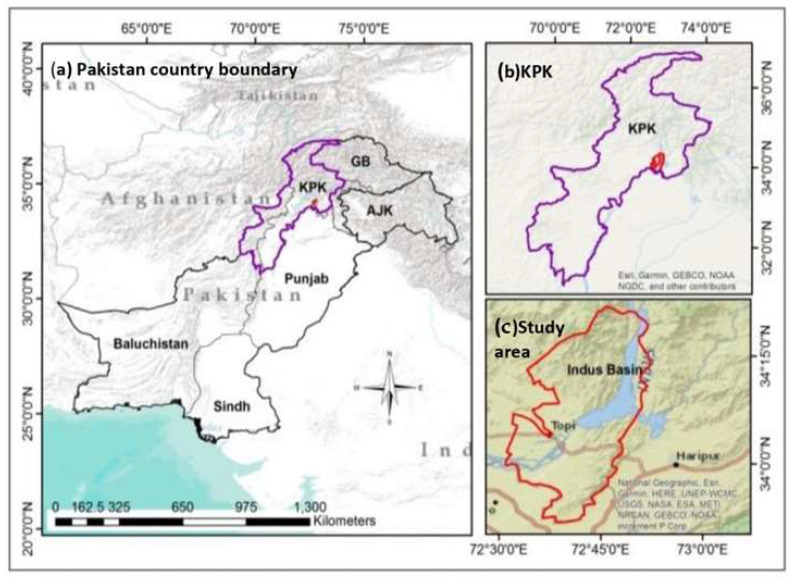
Study area map (Lower Mountainous Indus Basin area Terbella, Pakistan) (**a**) Country, (**b**) Province, and (**c**) Study area Terbella.

As given in Figure 1, the Terbella is situated between the Indus and Kabul River with geographic coordinates as 34°15′ N, 72°45′ E. The world’s largest earth dam is Terbella Dam, lying in the Indus Basin; it covers an area of 169,650 km^2^. It is located between the great Karakoram and the Himalayan glaciers mountain ranges, where over 90% of this region’s melt waters reach Terbella. The study area spreads about 1643 km^2^ residing overall population of 1,624,616 million [39]. Its boundaries are shared with districts of Buner in the north, Haripur in the east, Attock in the south and Mardan in the west. It is about 70 km at distance (northwest) from the capital of Pakistan i.e., Islamabad. The highest elevation is about 7000 feet above sea level. The mean temperature and average rainfall annually are 28.2 °C and 639 mm, consequently. The range of the monsoon season starts from May to October while the rainy winter term prevails from December to April. July is considered the hottest month which mean temperature 38 °C while January is the coldest month, with temperatures hitting about 10 °C [40]. The month of October is known as summer–winter phase due to seasonal changes. The climate of the area demonstrates temperature variations because of its inland position.

### 2.2. Remotely Sensed Data

The remotely sensed Landsat imageries were collected from USGS, NASA website with specific gap of 15 years, for example 1987, 2002 and 2017, respectively, for 30 years to assess the LULC and LST changes. The required images were taken over the span of 5 days (date 19–24) in the month of May. The images were radiometric and atmospherically corrected using Fast Line-of-sight Atmospheric Analysis of Spectral Hypercubes (FLAASH) method in Envi 5.3 software [23]. The imageries were proportionate for ecological variables such as temperature, humidity, scene ID and cloud cover in percentage and their details are available in Table 1.

### 2.3. Data Processing and Analysis

Pre-processing was used for satellite data before LULC classification and LST retrieval. Pre-treatment contains radiometric calibration, atmospheric corrections and line removal in QGIS, ArcMap and method of Support Vector Machine (SVM). The ENVI software was also used for LULC maps of different years (1987, 2002 and 2017) while the LST was measured from the derived thermal bands of the particular images [41].

### 2.4. LULC Classification and Accuracy Assessment

**Figure 2:**
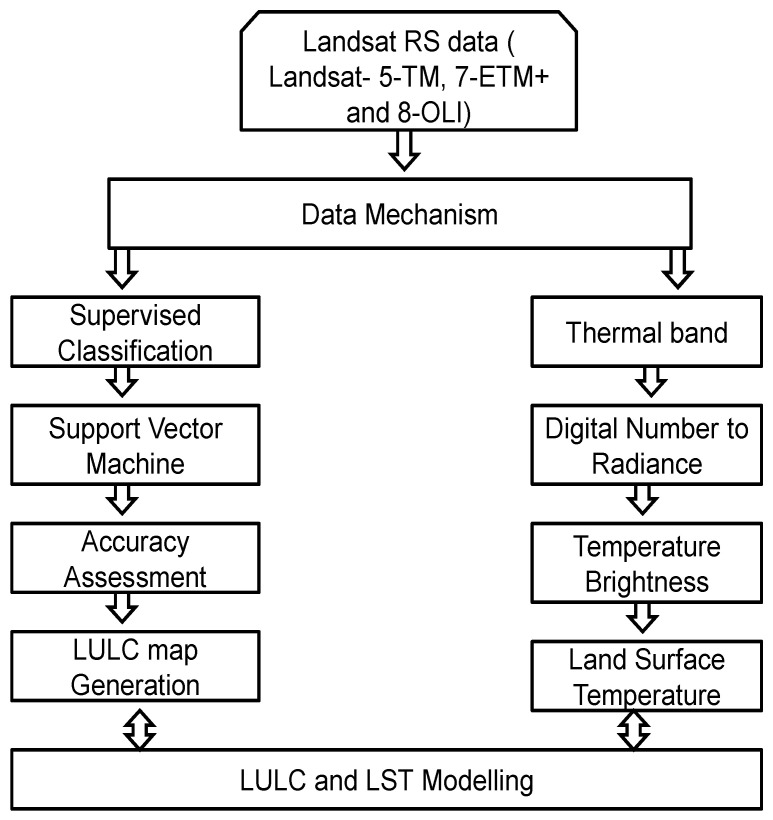
Flow chart for Land Use Land Cover and Land (LULC) and Land Surface Temperature (LST) methodology.

The Anderson classification method was used for the LULC grouping [42,43]. The Landsat-acquired images were used for LULC classification using Support Vector Machine(SVM) method in ENVI 5.3 software [44]. A total of 40 training ground truth samples from each LULC classes collected during the field survey were used to evaluate the LULC classification’s accuracy. During the field survey, the GPS system was used to gather the points. The training samples were created using spectral, spatial, and other data from Google Earth images in order to improve the accuracy of LULC classification. The LULC classification accuracy was frequently evaluated using the confusion matrix approach. The accuracy of LULC classification was measured using confusion matrix method. The confusion matrix method produced Kappa coefficient values, which were used for assessing the LULC classification accuracy [44,45]. The details of stepwise methodology are given in the Figure 2 which are as under.

### 2.5. LST Estimation

The Landsat satellite images were derived for LST from the geometrically and radiometrically corrected thermal bands. By applying standard RTE method, LST was collected from Landsat images of thermal bands the 5-TM and 7-ETM+ [46]. The Percentage Vegetation (PV), Normalized Difference Vegetation Index (NDVI), Surface Emissivity of Land applied in RTE technique (LSE). Equation (1) was applied for the NDVI calculation formula:

NDVI = NIR + RED/NIR − RED(1)

In the above equation, NIR stands for near-infrared band (band 5) while RED indicates the band red in (band 4) in Landsat-8 OLI. On the other hand, band 4 and 3 indicated the NIR and RED in Landsat-5 TM (0.64–0.67 mm) whereas band-4 (0.85–0.88 mm) in Landsat-8 OLI were somewhere constant with band-4 (0.77–0.90 mm) and band 3 (0.63–0.69 mm) in Landsat-5 and used for same results. Equation (2) was applied to examine PV that relied on NDVI minimum and maximum values.

PV = (NDVI − NDVI_min_/NDVI_max_ − NDVI_min_)^2^ (2)

LSE is necessary for LST retrieval; that is, the measurement part and maintaining the radiance of a black body (Planck’s Law) to predict discharge radiance [46]. Third equation was applied to calculate LSE:

LSEBi = Es (1 − FVC) + Ev FVC(3)

In equation, Bi indicates the number’s band and C indicates the rough surface(C-0 for plain surface) with regular significance of 0.005 while Es and Ev showed soil emissivity and foliage standards. The values were fixed at 0.971 and 0.987 for Es and Ev band 10 and 0.977 and 0.989 for band 11, respectively [47]. There is an adequate method for LST retrieval from Landsat 7 and 8, which used to make the body heat of the thermal band bloom at sensor level. Moreover, brightness temperatures derived from thermal bands composed of radiance estimation rely on the impact of Digital Number (DN) by utilizing the (NASA) data center. The fourth equation was used from the DN of satellite data.

Li = RADIANCEMULT_Bi_ × DN + RADIANCEADD_Bi_ (4)

Li is sensor spectral radiance (m W cm^−2^ sr^−1^ µm^−1^); RADIANCEADD and RADIANCEMULT are stable bands, occurring in the header file. From the fifth equation, we assessed the temperature vividness.

Ts_Bi_ = K2_Bi_/log ((K1_Bi_/L_i_) +1) − 273.15 (5)

In this equation, Ts denotes temperature brightness in band I in Kelvin whereas K1 and K2 are stable. The technique was conducted by USGS to assess brightness temperature to calculate the LST from Kelvin to Celsius assumption: 273.15 from the findings [48]. Estimating LST, spectral radiance and TOA must be authentic to gain radiance spectral surface. Moreover, the atmospheric affect is also very crucial to study the temperature [42]. In the present research, we used an appropriate RTE technique suggested by [49] given in Equation (6).

LST_RTEBi_ = EiTi + ((1 − Ei) Down welling) + Upwelling (6)

In this equation, E_i_ shows the emissivity surface of one band, Ti denotes the radiance spectral, while upwelling and down welling are path radiance. To calculate the down welling & upwelling the 5.0 MORTON radioactive transforms code using US strong atmospheric profile by selecting the Urban Aerosol Model. Base radiance (Ti) assessed by applying the law of Planck:

Ti = C1/Wavelength 5 _Bi_ (exp (C2/wavelength _Bi_ × Ts) − 1) (7)

Mentioned in the equation, C1 andC2 are Planck radiation stables (C1 is 1.19104 × 108 Wµm4 m-2 sr-1 and C2 is 14,387.7 µm k), wavelength denoted band (band-10 = 10.602 and band 11 = 12.511), whereas Ts denotes the surface temperature derived from Equation (5). RTE technique was used for single bands (10 and 11). The sixth equation results for every band were used in equation seven to assess the average surface temperature of land. We applied thermal bands 6 and 10 of Landsat-5 and 8 images because of supplementary values to assess LST outcome. Band 11 was overlooked because of LST error assessment and water vapor absorption effect proposed in the earlier studies [5].

### 2.6. LST Change Relative Detection

To correlate the impact of LULC on the thermal environment, relative LST was used for the years 1987, 2002, and 2017. Participation of relative LST from LULC (decreased/increase) changes prevailed; the mean LST of a particular region through every pixel value is given in Equation (8):

RLST_ij_ = LSTij − LSTi mean (8)

RLSTij denotes the temperature pixel of j of class I; LST expresses the temperature cell j of class i, and LSTi indicates the average LST value for landscaping. When RLSTji is greater than 0, the pixel shows +ve contribution of configuration of LULC. When RLSTji is lesser than 0, then it shows –ve impact thermal circumstances [50,51].

### 2.7. Standardization of LST

When LST maps were produced for distinct 3 years and then they were normalized prior to further study. The 2017 LST image was made as a base to guide this standardization method. So, the Z-score approach was used as in [52], as:

LST_nj_ = (LST − LST_J_/LST_σj_) LST_σi_ + LST_i_ (9)

This shows that the LST_nj_ is the pixel-particular standardized LST for the mentioned years (1987 or 2002); while LST_J_ is the average image-proper values of the LST initial image proceeding to standardization. LSTJ is the normal image-particular LST-values for 1987 or 2002; LST_σj_ is the standard deviation of the particular image for the values of LSTfor 1987 and 2002, whereas LST_σj_ is the standard deviation for the base year (2017); and finally LST_i_ is the average image value of LST for the year 2017.

### 2.8. Zone-Wise Temperature Classification

The LST were divided into various zones: <15 °C; 15 to <21 °C; 21 to <24 °C; 24 to <27 °C; 27 to <30 °C; 30 to <35 °C; and =>35 °C. Evaluations were made possible to quantify the ratio of area under every temperature zone. The greatest temperature zone was set as equal to or above 35 °C whereas the lowest considered was less than 15 °C.

LST_s_ = LST − LST_u_/LST_Ω_ (10)

where LST_s_ = Standardized LST; LST_u_ = Mean LST calculated from 1987, 2002, and 2017; LST_Ω_ = LST standard deviation calculated from 1987, 2002, and 2017.

### 2.9. LULC Simulation

There are several methods which were used for modeling LULC changes, utilizing driving variables and past changes [53]. For the current study, integrated CA-ANN model was used for LULC simulation in QGIS 2.8 software [54]. The CA-ANN model was selected for LULC simulation to the current study because of its high accuracy [23]. Initially, it forecasted the ANN-transformation potential matrix and then simulates prospective LULC alterations by utilizing CA model in the MOLUSCE tool in QGIS. The CA model is an appropriate model to wrap the dynamic and static characteristic of LULC trends, which were applied to forecast land cover changes due to clear precision [24]. Different variables such height and space from the main roads and slopes were used as features of land use.

The distance from the road was estimated by applying data vector of the particular region relating to space’s role in the software of ArcGIS. Firstly, the ANN method was used for training and modeling of past LULC changes then CA model have been applied to simulate LULC changes for the period of 2047 with the help of MOLUSCE tool in QGIS 2.8 software. The validation of the CA-ANN model is important; therefore, CA-ANN model was validated by comparing simulated LULC for 2017 with estimated LULC of 2017 using MOLUSCE QGIS validation.

### 2.10. LST Simulation

The foremost issue for public planners and administrators is the phenomena of global warming that increasing the LST in urban areas [55]. A multi-dimensional Artificial Neural Network method in MATLAB [35] was used to forecast and simulate upcoming LST alteration by using the past patterns in study area. Using Many-Layer Perceptron (MLP), ANN responds directly to parameter of network that enhances the changes in network model. The MLP algorithm is reliant on error correction—learning ideas. In MLP, once the system obtains a string, its phenomenon possibly produces a low-accuracy random result. The prediction of LST depends upon the record system from 1987 to 2017. The region was subcategorized into georeference grids 500 m × 500 m to display the point range of spatial units applying QGIS. This size of grid is preferred due to the lower area where properties of single point can have a crucial effect. The example data were utilized to construct ANN in MATLAB for simulation. Furthermore, latitude and longitude of the example spatial unit were used to increase the modeling effectiveness. The greater the input parameters, the more accurate the system model. The forecasting of LST results contained the construction of network, training of network, performance of group assessment, and forecasting. Mean Square Error (MSE), parallel coefficient (R), and standard measure the system assurance. The regression study yield produces an assessment of how the target data set describes the result of conclusion. When the significance is 1, then the output data sets are completely linked. On the other hand, it is very difficult to obtain a value of 1. The Graphic User Interface (GUI) was produced to examine the routine indication before network implementation and finally it was determined to be okay and saved for further prediction.

Numerous hidden layers were set based on MSE and R. The hidden layers are crucial as they permit the network to show non-linear behavior, which affects the results. A recent study was chosen with three hidden layers, where the first is the learning rate (µ), which was set at 0.1 and the decay time (β) was used to maintain it during the research. The hidden layers were chosen based on MSE and R and are crucial as they permit the network to show a non-linear attitude to affect the results. The typical decay rate at the level of 0–1 (0 < β) and the 0.9 decay rate was applied to enhance the understanding, which was expressed if the function error between past and present was rising and the β promoted the learning limit µ with division if it was falling, then multiplied it to reanalyze the µ. The indices of LULC were used for LST forecasting year 2047 [56]. After LULC indices derivation, correlation (bivariate) assessment between LST and indices were used to index, which has strong correlation with LST. The index NDVI indicated significant correlation with LST (p is lesser than 0.05) and the RMSE and R values were set 2.942 and 0.485, respectively. The selected index, such as NDVI with regression model, was then applied to simulate LST for the year 2047. The regression analyses were performed between LST and NDVI to create regression equation.

LST = 30.10 + (2.14 × NDVI) LST = 24.24 + (12.20 × NDVI)

Different indices were applied to calculate the LST shifts; NDBaI, NDVI, NDBI and UI which formulas are given in Table 2.

The NDVI index was rearranged so it could predict <15 °C, 15 to <21 °C, 21 to <24 °C, 24 to <27 °C, 27 to <30 °C, and equal to or above 30 °C. The chosen index for NDVI for the year 2017 was applied using the CA-ANN model to simulate the changes during 2017–2047. Lastly, for LST modeling for 2047, regression equation and NDVI index were performed.

## 3. Results

We identified the past LST and LULC changed pattern, and then simulated for the upcoming 30 years (i.e., 2047).

### 3.1. Previous Variations Patterns in LULC Classes

**Figure 3:**
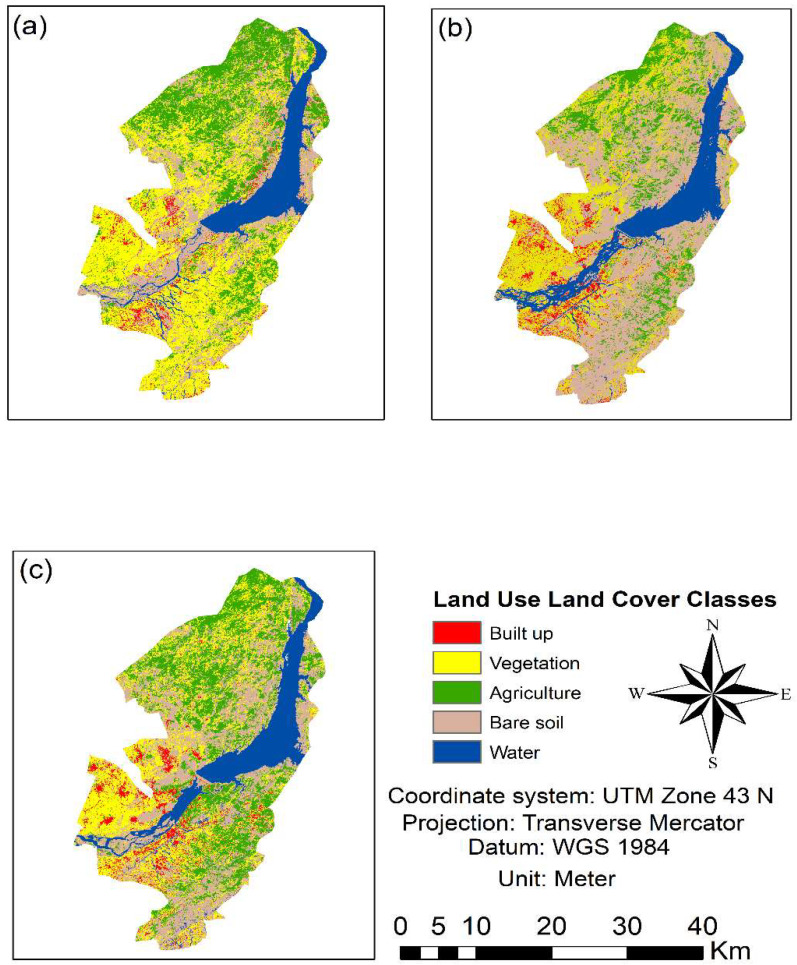
Past LULC maps of the years **a** (1987), **b** (2002), and **c** (2017).

**Figure 4:**
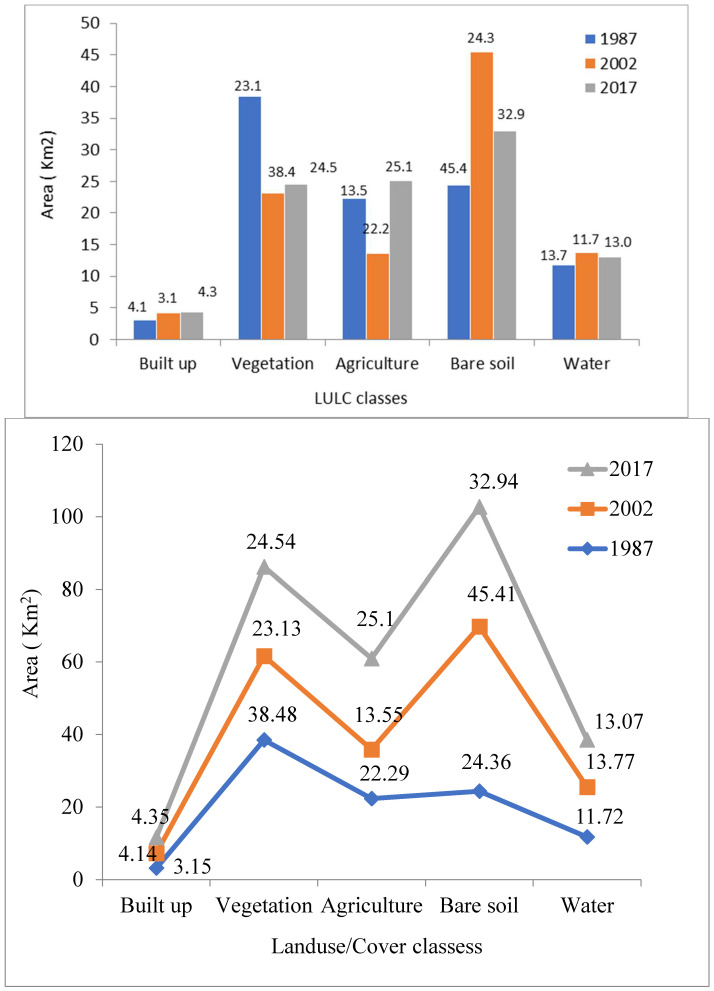
Classification of LULC classes during (1987–2017).

The entire study region was divided into five groups for the selected years. The results indicated that the built-up and bare soil types were 32 km^2^ and 251.2 km^2^ in 1987, while they increased to 44.9 km^2^ and 340.45 km^2^, respectively, in 2017. The vegetation showed a decreasing trend that covered an area of 396.76 km^2^ in 1987 and reduced to 253 km^2^ in 2017, as given in (Figure 3 and Figure 4). The LULC classification accuracy was above 90% for all three images, i.e., 1987, 2002, and 2017 (Table 3). Different factors may relate to LULC changes such as the rise in the built-up and bare soil due to anthropogenic activities and population explosion. Other reasons are natural disasters, due to which, for example, people from neighboring areas migrated to the studied area after the earthquake (2005). The Himalayan zone might be considered as urban area based on its population size and density.

### 3.2. Past Changes in LST (1987 to 2017)

**Figure 5:**
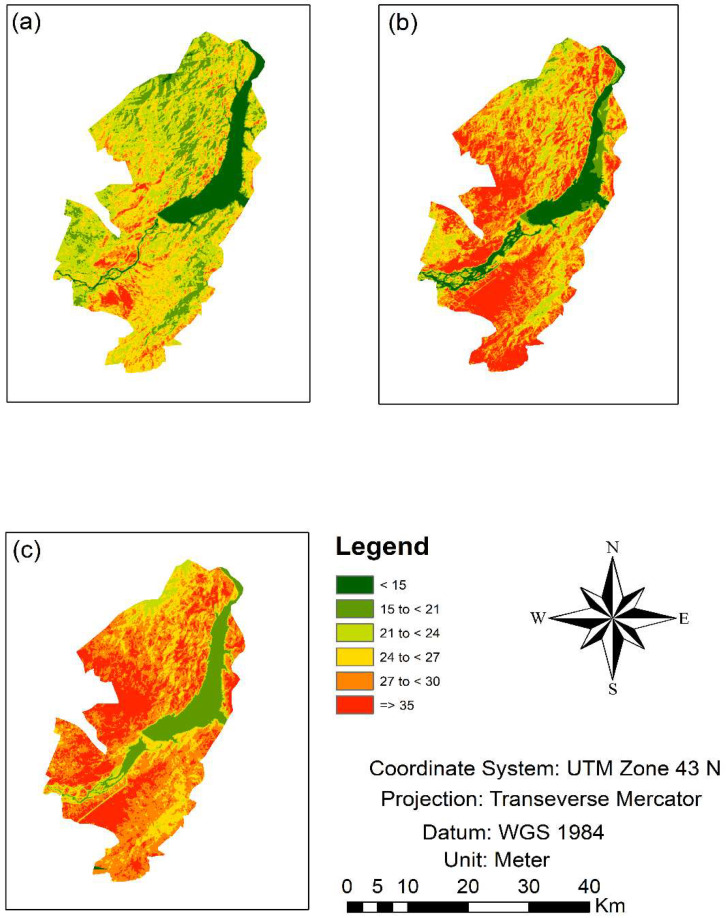
LST previous pattern for the respective years (**a**) 1987, (**b**) 2002, and (**c**) 2017.

**Figure 6:**
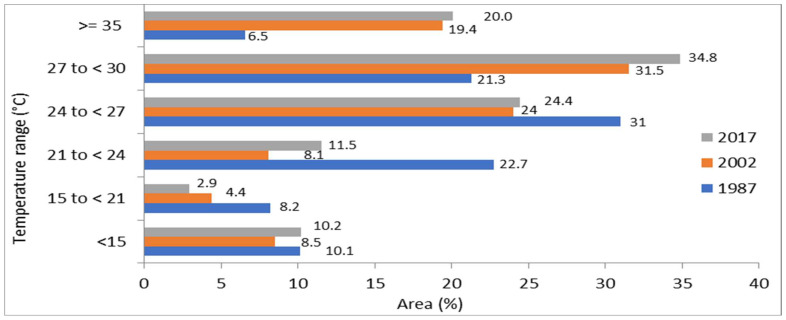
Distribution of different temperature ranges in area km^2^ for the years 1987, 2002 and 2017.

The study area images were classified into six major LST groups. The highest temperature range was set at 35 °C or above, while the lowest was considered 15 °C, which demonstrated the LULC shifts over different years, 1987, 2002, and 2017. The higher classes temperature area (27–30 °C and equal or greater than 35 °C) were 219.88 km^2^ and 67.51 km^2^ in 1987, which increased to 259.71 km^2^ and 200.31 km^2^ in 2017. On the other hand, the lower class in the range of <15 °C decreased from 104.85 km^2^ to 86.8 km^2^in area during the study period (Figure 5 and Figure 6). Mostly, a lower-temperature area changed into ahigher-temperature area due to changes in climate, urbanization, and indicated growing trends in the LST. The proposed study indicated that constructed and bare soils have greater LST than that of other classes such as water bodies, agriculture, and vegetation.

### 3.3. LULC Simulation for 2047

**Figure 7:**
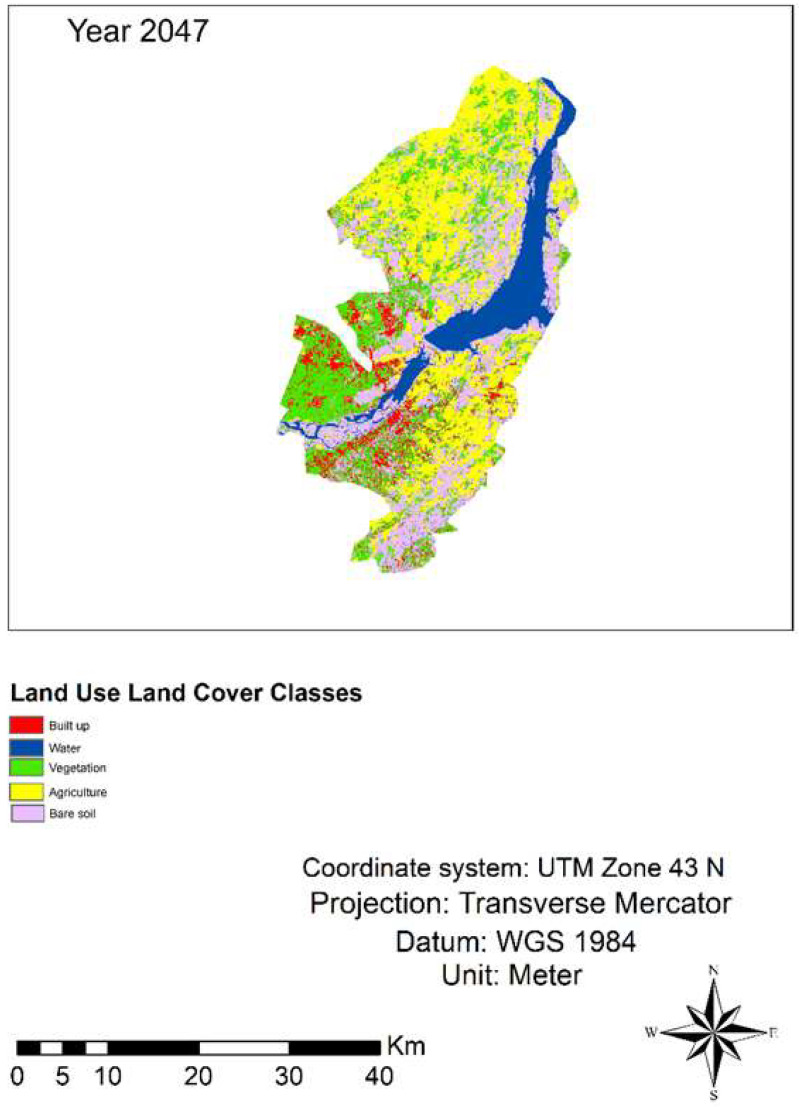
LULC simulation map for the year 2047.

The findings of the following studies indicated that the LULC changed from 1987 to 2017 and as well as for the projected year 2047. The projected outcome showed that the vegetation class was 253 km^2^ in 2017, which will decrease to 249 km^2^ in 2047 while built-up class will increase from 44.9 km^2^ to 70.08 km^2^, respectively, from 2017 to 2047 in the study area (Table 4 and Figure 7). The LULC modeling accuracy was assessed by percent correctness value which was above 70% (Table 5).

### 3.4. Simulation of LST for 2047

**Figure 8:**
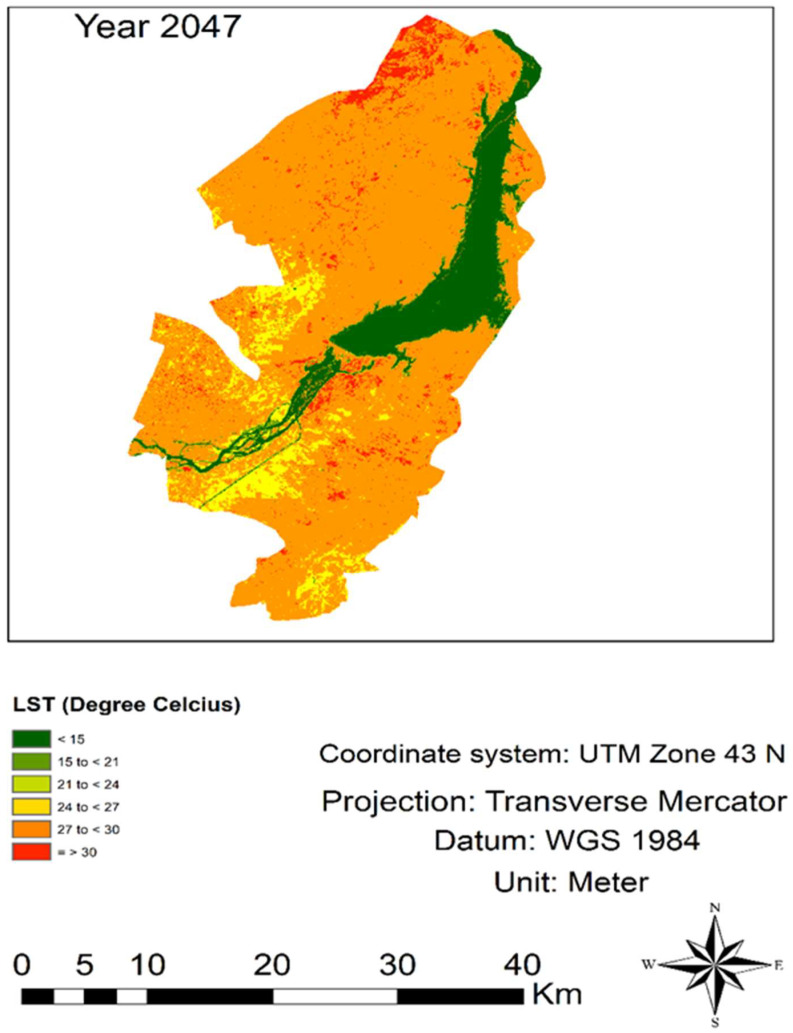
LST simulation map for the year 2047.

The LST was substantially altered in the study area, just like the LULC groups. Therefore, the LST was also simulated to assessed the strong correlation between the existing and forecasted accuracy prediction. Results indicated the increasing trend of higher-temperature series in range of, i.e., 27 to <30 °C, and the decreasing trend in the lower-temperature range (i.e., 21 to <24 °C) (Table 6 and Figure 8). This may be due to air temperature, global warming, and Urban Heat Islands (UHIs) affecting the LST of the study area (Table 6 and Figure 8).

## 4. Discussion

### 4.1. Past LULC Changes

Natural disasters might be chief reason for why an abundant number of people from neighboring areas left their region after the earthquake of 2005 in the western and eastern Himalayan areas, which may considered a remarkable source of urbanization. The consequences of such study supported the achievements of [57]. It was also reported in some other studies that economical, technological, geopolitical, and environmental parameters are responsible for urbanization [58]. Changes in the LULC are a major issue (FAO, 1999), and are the driving force of environmental changes. Rapid migration from rural areas to metropolitan areas causes instantaneous changes in the ecosystem of urban areas, biodiversity, universal landscaping, topography, and the biophysical environment [2]. There has already been available research in which it is mentioned that enlargement of LST is directly related to expansion of urban areas, especially in less-developed countries [59]. The metropolitan areas’ expansions have expressed an impact on the enlargement of bare earth surfaces [60,61]. Additionally, Past research has pointed out that vegetation land can cause soil moisture to recede LST [62].

### 4.2. Past LST Changes

The proposed study indicated that built-up and bare soils have a greater LST then that of other classes such as water bodies, agriculture, and vegetation. Mostly lower-temperature areas changed into higher-temperature zones because of climate change, urbanization, and the indicated growing trend in the study area’s LST. The same findings have been reported by different researchers in previous studies [19,63]. Furthermore, [64] also stated that heat discharge from the Himalayan plateau is a significant point of precipitation, as temperature variation is produced between the air at upper and lower elevations, but it has less impacted at lower-altitudinal regions, such as our study area [65]. The warm insulation and humid air between the Polar and subtropic region is one more significant factor that affects the Himalayan area’s climate. Current studies are focused on LULC changes (rather than climate changes) as local heating impacts of air temperature in the lower mountainous areas, which consequently shifts the temperature and moistening of air in the boundary area [66].

### 4.3. LULC Simulation

The past LULC trends indicated a drastic alteration in the proposed area during 1987 to 2017, which is why simulation of LULC dynamics is very significant for upcoming time. However, if the results remained the same as they were in the past, it would directly or indirectly affect the biodiversity and microclimate of the study area [67]. The CA-ANN model precision indicated the accuracy value of 70%, considered a permissible limit of accuracy. The forecasted result showed the decreasing trend in vegetation and increasing trends in the built-up and bare soil areas. Such a finding, also reported by [15] in Beijing, informs us that a population bomb is the main cause for intensifying these changes. Precedent management is needed to protect the study area from drastic alteration, as 49.91% of people live in urbanized areas, and this is estimated to reach 60% of people worldwide by 2030. In addition, the number of large cities will reach to 100 by 2025 [4].

### 4.4. LST Simulation (2047)

The projected results indicate the rising drift (409 Km^2^) in the higher-temperature zone (i.e., 27 to <30 °C), whereas the lower-temperature zone in range of (i.e., 12 to <21 °C) showed a decreasing trend, which is (42 Km^2^). Several elements such as global warming, greenhouse gases, and changes in surface features directly or indirectly affect the LST of the area [68,69]. Similarly, the expansion in built-up areas may also be the reason for the expansion in temperature [70]. The LST expansion badly affects the land cover thermal command that leads to UHIs from land surface changes that graciously left heat from anthropogenic sources [71]. The phenomena specifically increase the urban temperature [72], which is the foremost ecological problem for humans and the variety of life forms [73]. The warming trend in South Asia is probably greater than the global mean [74]. Additionally, the effect of global warming could be measured by land cover area close to construction [75,76].

## 5. Conclusions

A variation in LST related to LULC changes affected the normal functions of the ecosystem. This study found significant changes in LULC, especially in the area covered by built-up and bare soil, which has influenced the LST. The LST variations related to the physical properties of the surfaces, such as built-up and bare soil, have a greater contribution in increasing LST, while water and vegetation keep the environment cool. The simulation outcome showed that this will continue by the year 2047, if the same trends remain as the past. Overall, the study findings provide significant insights for landscape planners to act for monitoring the unplanned urban development and related UHIs formations. The crucial alarm is about the recently built link road under the China Pakistan Economic Corridor (CPEC) project, and famous earth-filled dam in the Indus basin passing through the environmentally sensitive lower mountainous study areas that may have a bad effect on LULC features. Some important steps should be taken before conducting further studies.

The high-resolution satellite imageries such as (IKONOS-2, OrbView-3, and historical SPOT data) could be used for further studies.

The study findings could be used for better LULC planning as well as UHI mitigation in the study area.

The study could be more comprehensive by combining climate data with LULC and LST dynamics in the study area.

Future LULC and LST simulation should be done in 15-year of intervals for better understanding of LULC and LST changes.

## Figures and Tables

**Table 1:** Source origin of downloaded images(United States Geological Survey).

Collection Date	Path/Row	Cloud (%)	Sensors	Scene ID
24 May 1987	150/36	6	Landsat 5	TM LT51500361987114ISP00
19 May 2002	150/36	8	Landsat 7	ETM + LT71500362002139SGS00
20 May 2017	150/36	13	Landsat 8	OLI LC81500362017140LGN00

**Table 2:** Formulas for various LULC indices.

Indices name	Equations Landsat (TM, ETM +, and OLI)
NDVI	Near Infrared-Red/Near Infrared + Red
UI	SWR2-Near Infrared/SWR2+ Near Infrared
NDBaI	SWRI-Thermal Infrared/SWRI + Thermal Infrared
NDBI	SWRI-Near Infrared/SWRI+Near Infrared

**Table 3:** Accuracy assessment of the classified land cover maps for 1987, 2002, and 2017.

Year	User Accuracy (%)	Producer Accuracy (%)	Overall Accuracy (%)	Kappa Coefficient
1987	96.34	93.15	94.96	0.92
2002	96.34	86.24	92.26	0.88
2017	93.67	92.84	91.35	0.87

**Table 4:** Land use land cover condition projection for the year 2047.

Class Name	Area (km^2^) 2047	Area (%)
Built up	070.08	06.79
Water	120.27	11.65
Vegetation	249.32	24.16
Agriculture	288.26	27.93
Bare soil	303.92	29.45

**Table 5:** Validation of CA-ANN model for LULC changes for the year 2047.

Validation of CA-ANN Model in QGIS Software			
Validation Parameters (K Parameters) and % Correctness			
K location	K histogram	Overall kappa	% Correctness
0.60	0.98	0.59	71.60

**Table 6:** LST Simulation for the 2047.

Temperature Range	Area (km^2^) 2047	Area (%)
<15 °C	129.0	12.50
21 to <24 °C	09.80	00.95
24 to <27 °C	76.24	07.38
27 to <30 °C	68.80	74.50
≥30 °C	47.98	04.65

## Data Availability

The Landsat data for the suggested study was freely available in the online portal of USGS-NASA website.
